# Epidemiological and clinical characteristics of mpox cases: reply

**DOI:** 10.1590/S2237-96222023000100029

**Published:** 2023-04-03

**Authors:** Ana Roberta Pascom, Isabella Nepomuceno de Souza, Amanda Krummenauer, Magda Machado Saraiva Duarte, Janaina Sallas, Daniela Buosi Rohlfs, Gerson Mendes Pereira, Arnaldo Correia de Medeiros, Angelica Espinosa Miranda

**Affiliations:** 1 Ministério da Saúde, Secretaria de Vigilância em Saúde, Brasília, DF, Brazil

The letter published containing comments about the study entitled *Epidemiological and clinical characteristics of monkeypox cases in Brazil in 2022: a cross-sectional study* emphasizes the need for laboratory quality control and awareness of health professionals as success factors for the early identification of mpox.^1^ We take this opportunity to highlight that, in order to achieve the objective of comprehensive care in this context, we also consider public health surveillance, monitoring and research to be relevant. The purpose of the manuscript was to present the profile of mpox infection in Brazil from the point of view of surveillance, which goes beyond laboratory diagnosis and professional training, also considering the clinical and epidemiological aspects of reported cases. Its focus was on identifying trends, describing the disease pattern and documenting this health condition.^2^

Of the 33,513 notified cases, 27,776 (82.9%) had a record of a laboratory test having been performed and the test result was pending for 1,587 of them on the date of the study. Almost all confirmed cases (99.8%; 7,784) and discarded cases (99.2%; 17,891) had a laboratory diagnosis. Also, of the 456 cases that had inconclusive or indeterminate test results, eight were classified as confirmed and 112 as discarded, according to other epidemiological criteria established by the health surveillance service. It is worth noting that, indeed, difficulty in accessing laboratory diagnosis and the need to train health teams to identify cases and interpret results, are additional barriers to controlling the outbreak.

In Brazil, laboratory confirmation of mpox is done by molecular testing (q-PCR) followed by the sequencing technique, in accordance with the recommendations of the Brazilian National Health Surveillance Agency (Agência Nacional de Vigilância Sanitária - ANVISA). There are countless factors that can contribute to false-negative results, ranging from technical reasons to poor sample quality. ^3^ ^,^ ^4^

We recognize as legitimate educational actions in the health sector determined by the emergence of theoretical and methodological contributions, in the face of the appearance of new diseases, and we reiterate the importance of continuing professional training.^5^
